# Preconditioning L6 Muscle Cells with Naringin Ameliorates Oxidative Stress and Increases Glucose Uptake

**DOI:** 10.1371/journal.pone.0132429

**Published:** 2015-07-06

**Authors:** R. Dhanya, K. B. Arun, V. M. Nisha, H. P. Syama, P. Nisha, T. R. Santhosh Kumar, P. Jayamurthy

**Affiliations:** 1 Agroprocessing and Natural Products Division, National Institute for Interdisciplinary Science and Technology (NIIST), CSIR, Industrial Estate, Pappanamcode, Thiruvananthapuram-695019, Kerala, India; 2 Rajiv Gandhi Centre for Biotechnology, Thiruvananthapuram-695012, Kerala, India; Stellenbosch University, SOUTH AFRICA

## Abstract

Enhanced oxidative stress contributes to pathological changes in diabetes and its complications. Thus, strategies to reduce oxidative stress may alleviate these pathogenic processes. Herein, we have investigated Naringin mediated regulation of glutathione (GSH) & intracellular free radical levels and modulation of glucose uptake under oxidative stress in L6 cell lines. The results from the study demonstrated a marked decrease in glutathione with a subsequent increase in free radical levels, which was reversed by the pretreatment of Naringin. We also observed that the increased malondialdehyde level, the marker of lipid peroxidation on induction of oxidative stress was retrieved on Naringin pretreatment. Addition of Naringin (100 μM) showed approximately 40% reduction in protein glycation *in vitro*. Furthermore, we observed a twofold increase in uptake of fluorescent labeled glucose namely 2-(N-(7-Nitrobenz-2-oxa-1,3-diazol-4-yl)Amino)-2-Deoxyglucose (2 - NBDG) on Naringin treatment in differentiated L6 myoblast. The increased uptake of 2-NBDG by L6 myotubes may be attributed due to the enhanced translocation of GLUT4. Our results demonstrate that Naringin activate GSH synthesis through a novel antioxidant defense mechanism against excessive Reactive Oxygen Species (ROS) production, contributing to the prevention of oxidative damage in addition to its effect on glycemic control.

## Introduction

Diabetes mellitus is often defined as a hyperglycemic condition arising due to insulin resistance or impaired insulin secretion. The oxidative stress induced pathways is known to be associated with the onset of diabetes and its complications, which is the real cause of the morbidity and mortality associated with diabetes [1]. Among other factors, oxidative stress is also reported to be responsible for the impaired secretion of insulin and glucose utilization in peripheral tissues in diabetic conditions [2]. Under hyperglycemia, the inhibition of ROS generation or its neutralization is reported to prevent the development of diabetic complications [3–7]. Evans et al. [8] have also suggested the role of oxidative stress in generating insulin resistance by altering intracellular signaling pathways.

Antidiabetic drugs like metformin are known to ameliorate hyperglycemia by lowering carbohydrate absorption without direct effect on insulin resistance [9–11]. There is a need to look for more efficacious agents for the management of diabetes, owing to the side effects of currently available drugs. Antioxidant treatments have demonstrated beneficial effects in animal models of diabetes [12–14]. However, these effects were not translated in larger clinical trials [15, 16] suggesting the need for new and more efficient antioxidants, targeting multiple factors of diabetes and its complications. For this reason, a potent antioxidant having antidiabetic properties will be a promising candidate for the treatment of both diabetes and its complications. There has been an increase in the use of plant extracts or their active components for health care needs over the last few decades [17]. Among them, Naringin, a flavonone found in grapefruit, has been reported to exhibit various therapeutic and pharmacological properties including antioxidant, antimicrobial, cardioprotective and antimutagenic effects [18]. It has also been reported to decrease blood glucose levels in streptozotocin induced diabetic rats by increasing plasma insulin levels [19]. As pathogenicity associated with diabetes is believed to be due to oxidative damage to the tissues by oxygen free radicals, it is sensible to elucidate the detailed protective mechanism of this potent antioxidant. The aim of this study is to investigate the role of Naringin in glucose uptake and GLUT4 translocation in skeletal muscle cell lines, the major hallmarks in diabetes. In addition, oxidative stress markers, like lipid peroxidation, glutathione, and intracellular reactive oxygen species were assessed as a possible mechanism underlying the Naringin effects.

## Materials and Methods

### 2.1. Materials

Dulbecco’s modified Eagle’s media (DMEM), antibiotic-antimycotic mix, insulin, rosiglitazone, MTT (3-(4,5-dimethylthiazol-2-yl)-2,5-diphenyl tetrazolium bromide), Dichloro-dihydro-fluorescein diacetate (DCFH-DA), Naringin and Tertiary butyl hydrogen peroxide (TBHP) were purchased from Sigma—Aldrich Chemicals (St Louis, MO, USA); Monoclonal anti-GLUT4 antibody, secondary anti-mouse immunoglobulin (IgG; Fab specific)–fluorescein isothiocyanate (FITC) antibody produced in goat were purchased from Santa Cruz Biotechnology, USA; Foetal bovine serum (FBS) was purchased from Gibco-BRL (Auckland, NZ); Horse serum was purchased from PAN Biotech (Aidenbach, Germany); 2-(7-Nitrobenz-2-oxa-1,3-diazol-4-yl) amino-2-deoxy-D-glucose (2-NBDG) was purchased from Molecular Probe (Invitrogen Life Technologies, Carlsbad, CA, USA); BCA protein assay kit was procured from Pierce Biotechnology, Rockford, USA; All other chemicals used were of standard analytical grade.

### 2.2. Cell culture and treatment

L6 myoblast cell line originally isolated from primary cultures of rat thigh was obtained from National Centre for Cell Sciences (NCCS), Pune, India. Differentiated L6 myoblast cell line is the best characterized cellular model of skeletal muscle origin to study glucose uptake and GLUT4 translocation. L6 myoblasts were maintained in DMEM supplemented with 10% FBS, 10% antibiotic-antimycotic mix at 37°C under 5% CO_2_ atmosphere. Cells were grown at a density of 1x10^4^ cells/well on 96-well black plates (BD Biosciences, Franklin Lakes, BJ) and 12-well plates (Costar, USA) for the immunofluorescent staining study and glucose uptake assay, respectively.

### 2.3. Cell viability

Viability of L6 myoblast was measured by means of MTT assay. Cytotoxicity of TBHP and Naringin were standardized based on both concentrations as well as period of incubation. Briefly, cells after incubation with TBHP (1, 10 & 100 μM) and Naringin (1, 10 & 100 μM) were washed and MTT (0.5 g/L) was added to each well for the estimation of mitochondrial dehydrogenase activity as described previously by Mosmann et al [20]. After 4 h incubation, 10% SDS in DMSO was added to each well and the absorbance at 570 nm of solubilized MTT formazan products were measured after 45 min using a microplate reader (BIOTEK-USA). Results were expressed as a percentage of cytotoxicity.

### 2.4. Measurement of Intracellular reactive oxygen species

Intracellular ROS was estimated by using DCFH-DA following method of Cathcart et al. [21]. Cells were induced with different concentrations of TBHP (1, 10 & 100 μM) to generate oxidative stress, Pretreatment of Naringin was carried for 3 and 24 h respectively. After washing with phosphate buffer saline (PBS, pH-7.4) cells were treated with DCFH-DA (20 μM) for 20 min. The cells were imaged after suspending in PBS using fluorescent microscope (Pathway 855, BD Bioscience, USA) equipped with filters in the FITC range (Excitation, 490 nm; and Emission, 525 nm). The fluorescent intensity was analyzed by BD Image Data Explorer software.

### 2.5. Lipid peroxidation

Lipid peroxidation was analyzed in L6 myoblast by thiobarbituric acid method [22]. After pretreatment, the cells were lysed and centrifuged at 13,000g for 2 min. Tthiobarbituric acid (1ml of 0.67%) dissolved in 5% trichloroacetic acid (TCA) was added to the supernatant. The mixture was heated at 100°C for 15 min. The absorbance was read at 532nm after cooling. Lipid peroxidation was expressed as nanomoles of malondialdehyde (MDA) per million cells, using the MDA extinction coefficient of 156 mM^-1^cm^-1^.

### 2.6. Determination of Intracellular glutathione concentration

Hissin and Hilf method was followed to determine reduced GSH levels in the cells [23]. Briefly, cells were lysed with metaphosphoric acid and the supernatant thus obtained was mixed with 100μl opthaldehyde along with 1ml of phosphate buffer saline. After 15 min, fluorescence was determined at excitation wavelength of 360 nm and emission wavelength of 460 nm. Results were expressed as nanomoles per mg of protein.

### 2.7. *In vitro* antiglycation assay

To determine the effect on *in vitro* glycation of protein, 500 μl of albumin (1 mg/ml) was incubated with 400 μl of glucose (500 mM) in the presence of 100 μl of Naringin at 1, 10 &100 μM, respectively. The reaction was allowed to proceed at 60°C for 24 h and 10μl of 100% TCA was added to stop the reaction. The mixture was centrifuged at 10000 g. Precipitate obtained was dissolved in 500 μl alkaline phosphate-buffered saline (PBS) (pH 10), and the fluorescence was measured at 370 nm (excitation) and 440 nm (emission) [24]. Ascorbic acid serves as a positive control.

### 2.8. Fluorescence analysis of 2-NBDG uptake by flow cytometry

Glucose uptake by active concentration of Naringin was confirmed by Flow cytometry analysis. Briefly following pretreatment culture medium was removed from each well and replaced with fresh culture medium in the absence or presence of 10 mM fluorescent 2-NBDG and incubated for 30 min. The cells were then washed twice with cold PBS, trypsinized, resuspended in ice-cold PBS and subjected to flow cytometry. Samples were analyzed using BD FACS Aria II (BD Biosciences) at FITC range (excitation 490 nm, emission 525 nm band pass filter). The mean fluorescence intensity of different groups were analyzed by BD FACS Diva software and corrected for autofluorescence from unlabeled cells.

### 2.9. Immunofluorescence staining

To investigate the molecular mechanism of the induction of glucose uptake GLUT4 upregulation was monitored by Laser based confocal imaging. After pretreatment with the Naringin (100 μM) and TBHP, cells were washed with PBS and fixed for 5 min with 4% formaldehyde and permeabilized with triton-X for 10 min. Cells were blocked with 5% BSA for 1 h followed by incubation with monoclonal GLUT4 antibody solution (1:200 dilution in 1.5% BSA in PBS) at 4°C overnight. And then incubated with FITC-conjugated goat anti-mouse IgG secondary antibody (1:500 dilution, 1.5% BSA in PBS) for 1 h. The cells were also counter stained with nuclear stain (DAPI- (4',6-diamidino-2-phenylindole)). Images were acquired using Laser Scanning Confocal microscope (Nikon A1R, Nikon Instruments, Melville, USA) equipped with filters in the FITC range (i.e. excitation, 490 nm; and emission, 525 nm). Images were analyzed by NIS ELEMENTS software.

### 2.10. Statistical analysis

Results were expressed as means and standard deviations of the control and treated cells from triplicate measurements (n = 3) of three different experiments. Data were subjected to one-way ANOVA, the significance of various groups were calculated by Duncan’s multiple range test using SPSS for Windows, standard version 16 (SPSS, Inc.) and significance was accepted at P≤0·05.

## Results

### 3.1. Cell viability

**Fig 1:**
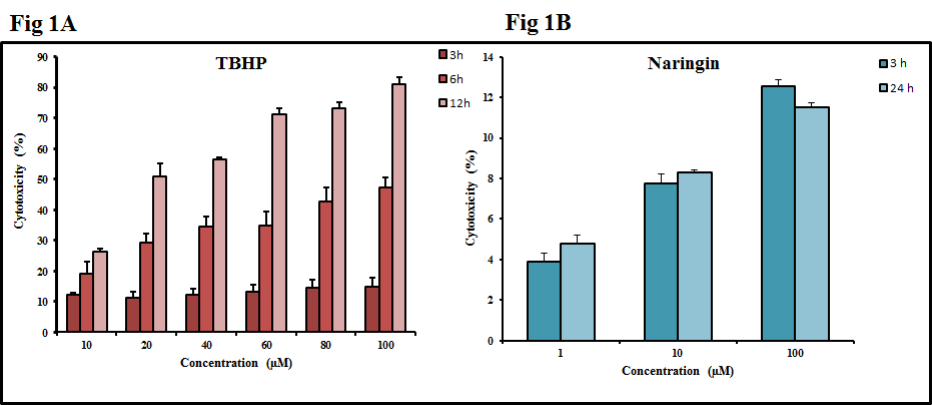
Cytotoxicity in cultured L6 myoblast. (A) Effect of TBHP on cell viability was evaluated based on concentration as well as period of incubation. (B) Cytotoxicity of Naringin was determined after 24 h preincubation in L6 myoblast. Each value represents mean ± SD (standard deviation) from triplicate measurements (n = 3) of three different experiments.

The cytotoxicity of TBHP was standardized based on concentration as well as period of incubation. TBHP and Naringin at 100 μM was found to be less than 20 (Fig 1A) and 15% toxic (Fig 1B) for a period of 3 and 24 h respectively. These concentrations were taken for further studies.

### 3.2. Determination of intracellular ROS

**Fig 2:**
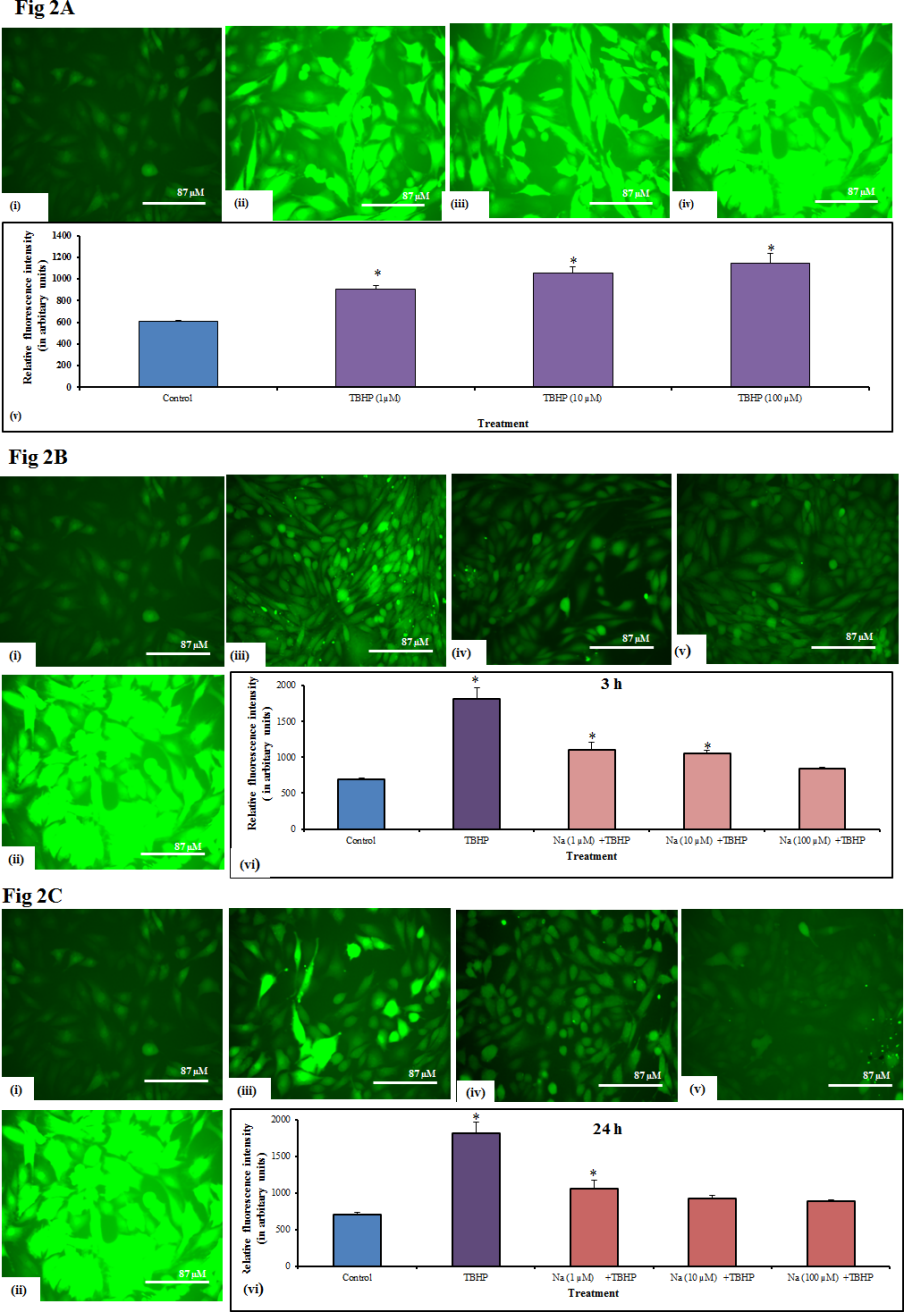
Intracellular ROS production and Fluorescence intensity analysis in L6 myoblast. (A) Fluorescence images (20 X magnifications) of untreated cell (i); Figures (ii), (iii) & (iv) represents cells induced with TBHP at 1, 10 & 100 μM; (v) Represents the relative fluorescence intensity analysis by BD Image Data Explorer software. Significance test between various groups was determined by using one way ANOVA followed by Duncan’s multiple range test. *P≤0.05 versus control. (B) Figure (i), (ii), (iii), (iv) & (v) represents fluorescence images of untreated cells, cells induced with TBHP (100 μM) and cells pretreated with Naringin (1, 10 & 100 μM) for 3h respectively; (vi) Represents the relative fluorescence intensity analysis by BD Image Data Explorer software. Significance test between various groups was determined by using one way ANOVA followed by Duncan’s multiple range test. *P≤0.05 versus control; #P≤0.05 versus TBHP.(C) Figure (i), (ii), (iii), (iv) & (v) represents fluorescence images of untreated cells, cells induced with TBHP (100 μM) and cells pretreated with Naringin (1, 10 & 100 μM) for 24h respectively; (vi) Represents the relative fluorescence intensity analysis by BD Image Data Explorer software. Scale bar corresponds to 87 μM. TBHP: Tertiary butyl hydrogen peroxide; Na1+TBHP, Na2+TBHP & Na3+TBHP represents relative fluorescence intensity analysis of cells pretreated with Naringin (1, 10 & 100 μM) followed by induction of TBHP; Each value represents mean ± SD (standard deviation) from triplicate measurements (n = 3) of three different experiments. Significance test between various groups was determined by using one way ANOVA followed by Duncan’s multiple range test.* P≤0.05 versus control; #P≤0.05 versus TBHP.

To investigate the effect of Naringin on oxidative stress associated with diabetes mellitus we induced stress in L6 skeletal muscle cells by using TBHP. Induction of free radicals with TBHP at three different concentrations (1, 10 & 100 μM) for 3 h revealed that cells generated significant levels of intracellular reactive oxygen species as compared to control (Fig 2A), (P≤0.05). TBHP at 100 μM (Fig 2A (iv)) showed a significant increase in intracellular ROS and this concentration was used to induce stress condition in further studies. Pretreatment of Naringin for 3 & 24 h at different concentrations (1μM, 10μM & 100 μM), dose dependently reduced ROS concentration as shown in Fig 2B & 2C (P≤0.05). The fluorescence intensity of the images was analyzed by BD Image Data Explorer software and has been illustrated in Fig 2A(v), 2B(vi) & 2C(vi).

### 3.3. Influence in lipid peroxidation

There was a significant increase in malonaldehyde concentration in L6 myoblast on induction of oxidative stress (0.513 nmol) than that of untreated control (0.253 nmol) (P≤0.05). Pretreatment with Naringin even at 1μM restricted the production of malondialdehyde to 0.39 nmol and Naringin at 10 & 100 μM showed a decrease to 0.36 & 0.29 nmol, respectively (P≤0.05) (Table 1).

**Table 1:** GSH, MDA levels in L6 myoblast after treatment in cultured L6 myoblast. The levels of GSH and MDA were assayed after treatment with Naringin followed by induction of oxidative stress in L6 myoblast. Each value represents mean ± SD (standard deviation) from triplicate measurements (n = 3) and the significance accepted at P≤0.05.

Treatment	Dose (μM)	MDA/10^4^cells (nmol)	GSH (nmol/mg of protein)	
		3h	3h	24 h
**Untreated control**	-	0.253±0.099	23.91± 2.13	
**TBHP**	100	0.513±0.019*	15.78 ±1.75*	
**Naringin**	1	0.39±0.088^#^	18.17±0.99^#^	20.10±1.07^#^
	10	0.361±0.078^#^	20.38±1.01^#^	25.20±0.97^#^
	100	0.29±0.055^#^	27.31±1.20^#^	27.70±0.49^#^

* P≤0.05 versus Control

^**#**^ P≤0.05 versus TBHP

### 3.4. Role in GSH metabolism

GSH levels were monitored after 3 and 24 h of Naringin (1, 10, and 100 μM) pretreatment to investigate the effect of Naringin in natural antioxidant defense system of L6 cells. Oxidative stress induced by TBHP reduced GSH level by 34% compared to control (P≤0.05). Naringin pretreatment retrived the GSH level to that of control as shown in Table 1.

### 3.5. Effect on Non-enzymatic glycation

**Fig 3:**
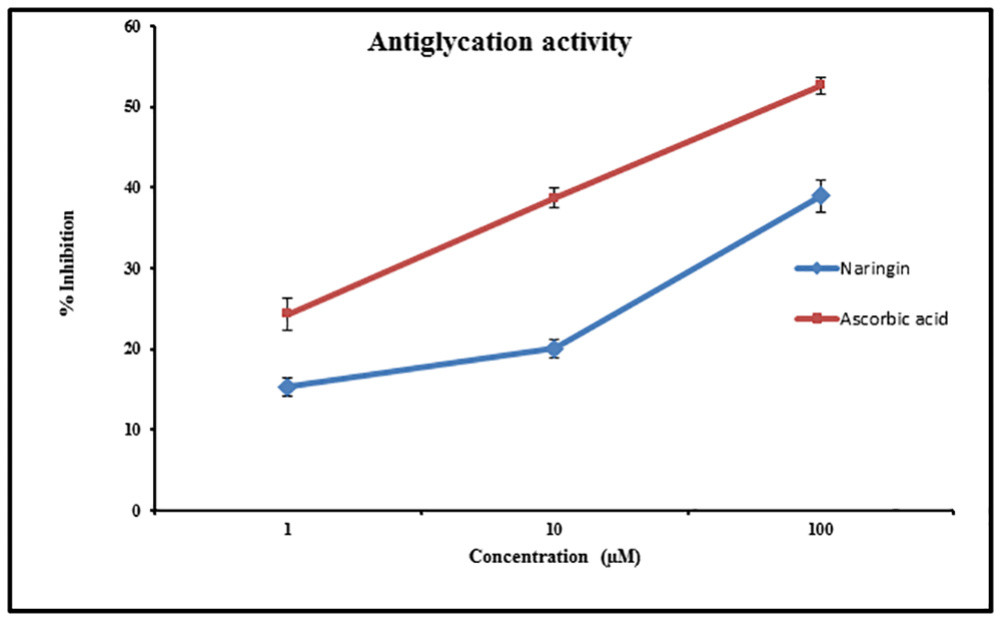
Antiglycation activity of Naringin. Antiglycation activity of Naringin at three different concentrations (1, 10 & 100 μM). Ascorbic acid was used as standard. Each value represents mean ± SD (standard deviation) from triplicate measurements (n = 3) of three different experiments.

Non-enzymatic glycosylation between reducing sugar and protein results in the formation of advanced glycation end products (AGEs), which is involved in diabetic complications. Thus, compounds that inhibit the formation of AGEs are believed to have therapeutic potentials in patients with diabetes. Naringin showed a marked decline in *in vitro* glycation of proteins in a dose dependent manner (Fig 3). Naringin at 100μM showed an inhibition of 40%. Ascorbic acid was used as a positive control (IC_50_ -30 μM).

### 3.6. Flow cytometric analysis of glucose uptake

**Fig 4:**
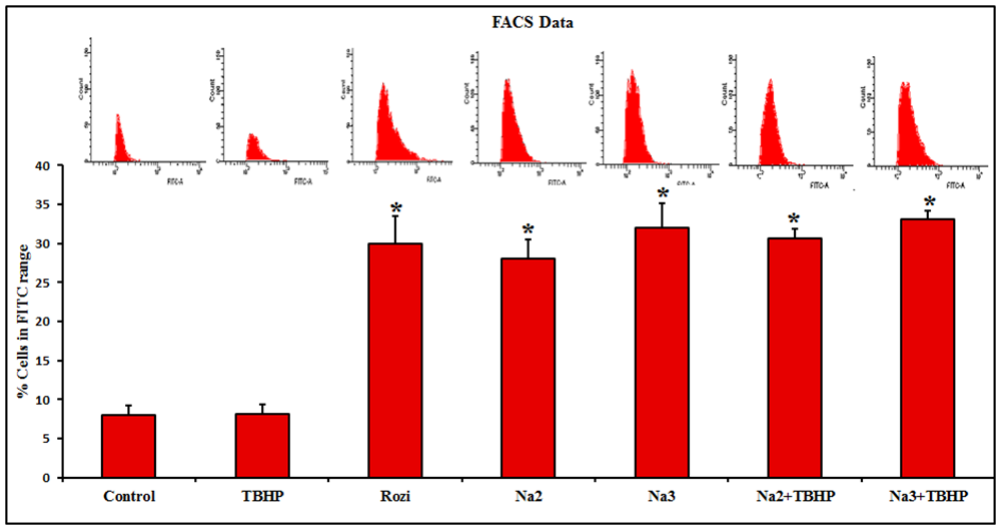
Fluorescence analysis of 2-NBDG uptake by flow cytometry. FACS analysis of 2-NBDG uptake in differentiated L6 cells by plotting cell count against FITC revealed that 8%, 8.1% and 30% of cells uptake 2-NBDG in control, TBHP and Rosiglitazone treated cells respectively whereas 30.6%, 33.1%, 28%, 32% of cells uptake 2-NBDG, pretreated with two different concentrations (10 and 100 μM) of Naringin along with/without TBHP respectively. Each value represents mean ± SD (standard deviation) from triplicate measurements (n = 3) of three different experiments. Significance test between various groups was determined by using one way ANOVA followed by Duncan’s multiple range test.* P≤0.05 versus control.

2-NBDG uptake in L6 myotubes was analyzed by flow cytometry by detecting the fluorescence within the cells. There was no significant effect on 2-NBDG uptake in the cell induced with TBHP (8.7%) compared to control (8%) (Fig 4). At 24 h pretreatment of Naringin (10 μM and 100 μm), the glucose uptake in L6 myotubes remarkably increased to 27.5% and 35.9%, which was much higher than that of positive control, Rosiglitazone (30.4%, P≥0.05).

### 3.7. Upregulation of GLUT4 in L6 myotubes

**Fig 5:**
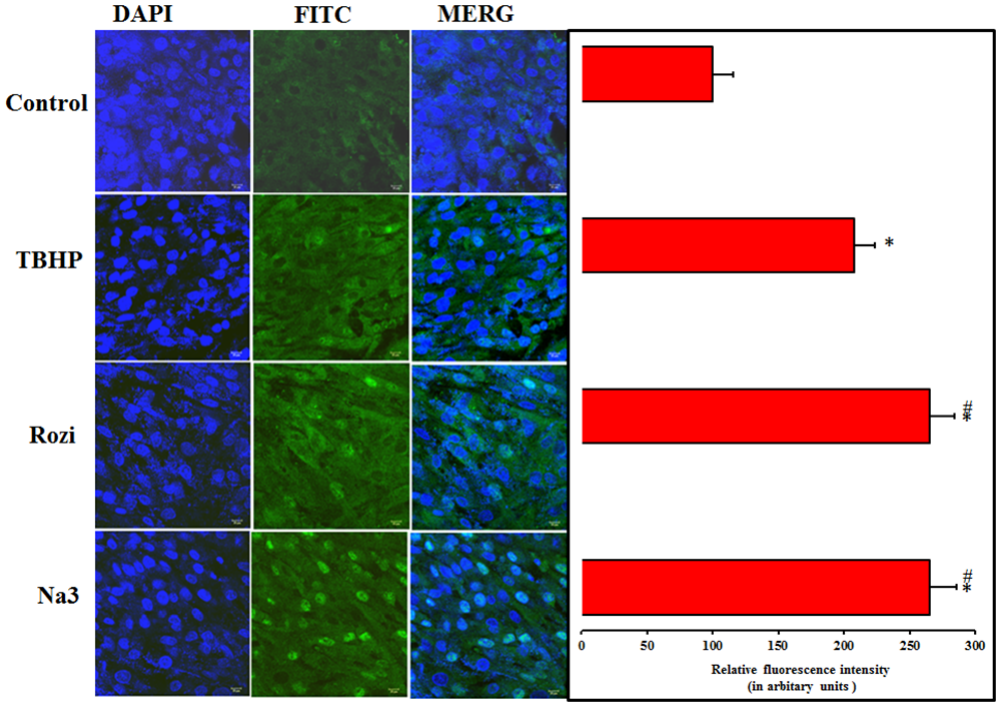
GLUT4 upregulation on Naringin pretreatment. Immunofluorescence assay visualized upregulation of GLUT4 in differentiated L6 myoblast. High resolution confocal images (40X) of Untreated L6 myotubes, L6 myotubes treated with TBHP, Rosiglitazone (100 nM) and Naringin (100 μM, 24 h). Scale bar corresponds to 10μM. Each value represents mean ± SD (standard deviation) from triplicate measurements (n = 3) of three different experiments. Significance test between various groups were determined by using one way ANOVA followed by Duncan’s multiple range test. * P≤0.05 versus control; #P≤0.05 versus TBHP.

Immunofluorescence staining revealed an upregulation of GLUT4 transporter protein on pretreatment of Naringin (100 μM) which was comparable to that of positive control as shown in Fig 5 (P≥0.05). The results obtained by quantifying immunologically labeled GLUT4 at the surface of intact cells correlates with that of glucose uptake, suggesting that the induction of GLUT4 by pretreatment of Naringin is likely responsible for enhanced glucose uptake exhibited by L6 myotubes. TBHP exposure to cells did not induce any changes in GLUT4 translocation.

## Discussion

Over the past decade, investigators had shown tremendous interest in studying the role of oxidative stress markers in the genesis of diabetes and its associated complications. Defective antioxidant system has been reported in diabetic patients compared to healthy subjects [25]. GSH, an abundant and ubiquitous antioxidant, function mainly as an efficient intracellular reductant, protecting cells from damage caused by free radical, drugs and radiation. Decreased GSH in TBHP treatment may be due to its increased utilization in protecting cells. In the present study, GSH was retrieved to normal levels in L6 myoblast on pretreatment with Naringin. GSH is an important substrate for glutathione peroxidase (GPx) and glutathione-S-transferease, which is an inevitable part of our antioxidant system. Naringin treatment is reported to upregulate the gene expression of GPx in high cholesterol fed rat [26].

Defects in the antioxidant defense system are in close association with induction of lipid peroxidation [27]. We have observed increased level of malondialdehyde, a byproduct of lipid peroxidation on induction of oxidative stress with TBHP. Chronic pretreatment of Naringin significantly decreased thiobarbituric acid reactive substances levels, and our results are in agreement with Rajadurai et al. [28] who reported a decrease in mitochondrial lipid peroxidation in Wistar rats on Naringin pretreatment. Naringin has also been reported to improve catalase and superoxide dismutase activities in the heart of isoproterenol induced rats [29].

AGE's and their derivatives are reported to contribute significantly to the pathogenesis of diabetes and elicit oxidative stress that elevates diabetic complications. In our study, a significant decrease in glycation of bovine serum albumin was found on coincubation of Naringin at 100 μM. A number of AGE inhibitors, including aminoguanidine, improved diabetic complications in both animal models and clinical trials but are reported to possess numerous side effects [30]. Thus, Naringin may be considered as a promising candidate to reduce AGE's formation owing to its lesser side effects. Though anti glycation activity of many of the flavonoids has been extensively studied, there are only a few reports on the anti glycation property of Naringin.

Several clinical trials have demonstrated that treatment with antioxidants, such as, vitamin E, vitamin C or glutathione, improved insulin sensitivity in insulin resistant individuals [31, 32]. In the present study, we observed a substantial increase in glucose uptake in L6 myotubes on pretreatment of Naringin (100 μM) which was much higher than that of the antidiabetic drug, rosiglitazone. In our previous study, we have made a comparative evaluation of antidiabetic potential of Quercetin and Rutin, which pointed to the role of chemical modification on the biological activities of the flavonoids [33]. Inducing glucose uptake in muscle cells can have a remarkable effect on plasma glucose level owing to its large proportion in human body. Although our primary focus is on skeletal muscle, it is possible that Naringin may increase glucose uptake in the liver and fat tissue in addition to the effect in muscles. Mahamoud et al. [34] reported that pretreatment of hesperidin and Naringin decreased the levels of glucose, glycosylated hemoglobin, MDA, nitric oxide, TNF-α and IL-6 in streptozotocin induced rats. Naringin is also reported to modulate hepatic glycolysis and gluconeogenesis [35]. In addition, we found that Naringin pretreatment induced translocation of GLUT4 from intracellular compartments to plasma membrane to the same extent than that of rosiglitazone pointing to the molecular end mechanism utilized by Naringin in inducing glucose uptake.

## Conclusion

Strategies to control oxidative stress have become a relevant pharmacotherapy in the treatment of micro and macrovascular diabetic complications. Antioxidant like naringin, acting on different molecular targets of diabetes appears to be promising in the management of both diabetes and its associated complications. Therapeutic potential of natural antioxidants needs to be explored further to reveal their effect on molecular signaling.

## References

[1] Topic Aleksandra, Milenkovic Marina, Snezana Uskokovic Markovic, Vucicevic (2010) Insulin mimetic effect of tungsten compounds on isolated rat adipocytes. Biol Trace Elem Res 134:296–306. 10.1007/s12011-009-8474-y 19644657

[2] Zatalia SR, Sanusi H (2013) The role of antioxidants in the pathophysiology, complications, and management of diabetes mellitus. Acta Med Indones 49:141–147. 23770795

[3] Atli T, Keven K, Avci A, Kutlay S, Turkcaper N, Varli M et al (2004) Oxidative stress and antioxidant status in elderly diabetes mellitus and glucose intolerance patients. Arch Gerontol Geriatr 39:269–75. 15381345 10.1016/j.archger.2004.04.065

[4] Osawa T, Kato Y (2005) Protective role of antioxidative food factors in oxidative stress caused by hyperglycemia. Ann N Y Acad Sci 1043:440–451. 16037265 10.1196/annals.1333.050

[5] Shen X, Zheng S, Metreveli NS, Epstein PN (2006) Protection of cardiac by overexpression of MnSOD reduces diabetic cardiomyopathy. Diabetes 55:798–805. 16505246 10.2337/diabetes.55.03.06.db05-1039

[6] Vincent AM, Russell JW, Sullivan KA, Backus C, Hayes JM, Mc Lean LL et al (2007) SOD2 protects neurons from injury in cell culture and animal models of diabetic neuropathy. ExpNeurol 208:216–227. 10.1016/j.expneurol.2007.07.017 PMC2190625 17927981

[7] Otero P, Bonet B, Herrera E, Rabano (2005) Development of atherosclerosis in the diabetic BALB/c mice. Prevention with vitamin E administration. Atherosclerosis 182:259–265. 16159598 10.1016/j.atherosclerosis.2005.02.024

[8] Evans JL, Goldfine ID, Maddux BA, Grodsky GM (2003) Are oxidative stress activated signaling pathways mediators of insulin resistance and cell dysfunction? Diabetes 52:1–8. 12502486 10.2337/diabetes.52.1.1

[9] Knowler WC, Barrett Connor E, Fowler SE, Hamman RF, Lachin JM, Walker EA et al(2002) Reduction in the incidence of type 2 diabetes with lifestyle intervention or metformin. N Engl J Med 346:393–403. 11832527 10.1056/NEJMoa012512 PMC1370926

[10] Buchanan TA, Xiang AH, Peters RK, Kjos SL, Marroquin A, Goico J, Ochoa C et al (2002) Preservation of pancreatic beta-cell function and prevention of type 2 diabetes by pharmacological treatment of insulin resistance in high-risk Hispanic women. Diabetes 51:2796–98. 12196473 10.2337/diabetes.51.9.2796

[11] Chiasson JL, Josse RG, Gomis R, Hanefeld M, Karasik A, Laakso M (2002) Acarbose for prevention of type 2 diabetes mellitus: the STOP-NIDDM randomized trial. Lancet 359:2072–2077. 12086760 10.1016/S0140-6736(02)08905-5

[12] Kowluru RA, Kenned A (2001) Therapeutic potential of anti-oxidants and diabetic retinopathy. Expert Opin Invest Drugs 10:1665–1676. 10.1517/13543784.10.9.1665 11772276

[13] Packer L, Kraemer K, Rimbach G (2001) Molecular aspects of lipoic acid in the prevention of diabetes complications. Nutrition 17:888–895. 11684397 10.1016/s0899-9007(01)00658-x

[14] Abiko T, Abiko A, Clermont AC, Brett S, Naoichi H, Junichi T et al (2003) Characterization of retinal leukostasis and hemodynamics in insulin resistance and diabetes: role of oxidants and protein kinase C activation. Diabetes 352:829–837. 10.2337/diabetes.52.3.829 12606527

[15] Beckman JA, Goldfine AB, Gordon MB, Andrea D, Marie Gerhard H, Mark AC (2003) Oral antioxidant therapy improves endothelial function in Type 1 but not Type 2 diabetes mellitus. Am J Physiol 285: H2392–2398. 10.1152/ajpheart.00403.2003 12881209

[16] Gaede P, Poulsen HE, Parving HH, Pedersen O (2001) Double-blind, randomized study of the effect of combined treatment with vitamin C and E on albuminuria in Type 2 diabetic patients. Diabetic Med 18:756–760. 11606175 10.1046/j.0742-3071.2001.00574.x

[17] Hilmi 1Y, Abushama FM, Abdalgadir H, Khalid A, Khalid H (2014) A study of antioxidant activity, enzymatic inhibition and *in vitro* toxicity of selected traditional sudanese plants with anti-diabetic potential. CAM 14:149. 10.1186/1472-6882-14-149 PMC4017226 24885334

[18] Jeon SM, Park YB, Choi MS (2004) Antihypercholesterolemic property of Naringin alters plasma and tissue lipids, cholesterol-regulating enzymes, fecal sterol and tissue morphology in rabbits. Int J Clin Nutr 23:1025–1034. 10.1016/j.clnu.2004.01.006 15380892

[19] Punithavathi VR, Anuthama R, Prince PS (2008) Combined treatment with Naringin and vitamin C ameliorates streptozotocin-induced diabetes in male Wistar rats. J Appl Toxicol 28:806–813. 10.1002/jat.1343 18344197

[20] Mosmann T (1983) Rapid colorimetric assay for cellular growth and survival: application to proliferation and cytotoxicity assays. J Immunol Methods 65:55–63. 6606682 10.1016/0022-1759(83)90303-4

[21] Cathcart R, Schwiers E, Ames BN (1983) Detection of picomole levels of hydroperoxides using a fluorescent dichlorofluorescein assay. Anal Biochem 34:111–116. 10.1016/0003-2697(83)90270-1 6660480

[22] Moore K, Roberts LJ (1998) Measurement of lipid peroxidation. Free Radical Res 28:659–671. 9736317 10.3109/10715769809065821

[23] Hissin PJ, Hilf R (1976) Fluorometric method for determination of oxidized and reduced glutathione in tissues. Anal Biochem 74:214–226. 962076 10.1016/0003-2697(76)90326-2

[24] Arom J (2005) *In vitro* antiglycation activity of Arbutin. J Naresuan University 3:35–41.

[25] Likidlilid A, Patchanans N, Peerapatdit T, Sriratanasathavorn C (2010) Lipid peroxidation and antioxidant enzyme activities in erythrocytes of type 2 diabetic patients. J Med Assoc Thai. 93:682–93. 20572373

[26] Jeon SM, Bok SH, Jang MK, Kim YH, Nam KT, Jeong TS et al (2002) Comparison of antioxidant effects of Naringin and probucol in cholesterol-fed rabbits. Clin Chem Acta 317:181–90. 10.1016/s0009-8981(01)00778-1 11814474

[27] Jagetia GC, Baliga MS (2003) Evaluation of the radioprotective effect of the leaf extract of *Syzygium cumini* (Jamun) in mice exposed to a lethal dose of gamma-irradiation. Nahrung 47:181–5. 12866620 10.1002/food.200390042

[28] Rajadurai M, Stanely Mainzen Prince P (2009) Naringin ameliorates mitochondrial lipid peroxides, antioxidants and lipids in isoproterenol induced myocardial infarction in Wistar rats. Phytother Res 23:358–362. 10.1002/ptr.2632 18844325

[29] Sathish V, Ebenezar KK, Devaki T (2003) Synergistic effect of nicorandil and modipine on tissue defense system during experimental myocardial infarction in rats. Mol Cell Biochem 243:133–8. 12619898 10.1023/a:1021612230000

[30] Thornalley PJ (2003) Use of aminoguanidine (Pimagedine) to prevent the formation of advanced glycation end products. Arch Biochem Biophys 419:31–40. 14568006 10.1016/j.abb.2003.08.013

[31] Ceriello A (2000) Oxidative stress and glycemic regulation. Metabolism 49:27–29. 10693917 10.1016/s0026-0495(00)80082-7

[32] Jie Jia, Zhang Xi, Hua Yong-Shan, Wu Yi, Qing Zhi Wang, Na-Na Li et al (2009) Evaluation of *in vivo* antioxidant activities of Ganodermalucidum polysaccharides in STZ-diabetic rats. Food Chem 115:32–36.

[33] Dhanya R, Arun KB, Syama HP, Nisha P, Sundaresan A, Santhosh Kumar TR, et al (2014) Rutin and Quercetin enhance glucose uptake in L6 myotubes under oxidative stress induced by tertiary butyl hydrogen peroxide. Food Chem 158:546–554. 10.1016/j.foodchem.2014.02.151 24731381

[34] Mahmoud AM, Ashour MB, Abdel-Moneim A, Ahmed OM (2012) Hesperidin and Naringin attenuate hyperglycemia mediated oxidative stress and proinflammatory cytokine production in high fat fed streptozotocin induced type 2 diabetic rats. J Diabetes Complications 26:483–490. 10.1016/j.jdiacomp.2012.06.001 22809898

[35] Leelavinothan P, Selvaraju S (2010) Efficacy of Naringin on hepatic enzymes of carbohydrate metabolism in streptozotocin nicotinamide induced type 2 diabetic rats. Int J Pharm Biol Arch 1:280–286.

